# Animacy, postpositions, and the spatial cases in Niya Prakrit

**DOI:** 10.1515/jsall-2025-0004

**Published:** 2025-06-09

**Authors:** Niels Schoubben

**Affiliations:** Leiden University Centre for Linguistics, Reuvensplaats 3–4 2311 BE Leiden, The Netherlands

**Keywords:** animacy, postpositions, ablative, locative, Niya Prakrit

## Abstract

In this paper, it is shown that (i) the synthetic ablative and locative case in Niya Prakrit have come to be restricted to inanimate nouns and pronouns, and that (ii) analytic formations with postpositions are used for animates: (pro)noun + *paride* ‘from’ for ablatival functions, and (pro)noun + *vaṃti* ‘in, at, to’ for locatival functions. In addition, it is argued that in the Khotan Prakrit document CKD 661 the postposition *sag̲aj̲i* /saγāźi/ ‘in, at, to’ is used in the same function as Niya Pkt. *vaṃti*.

## 1 Introduction and aims

The grammaticalisation of postpositions as new case markers is a well-known trait of New Indo-Aryan (NIA) languages (Masica 1991: 230–248), but precursors of this development can already be found in Middle Indo-Aryan (MIA) and Old Indo-Aryan (OIA).

Using a corpus of texts in Vedic Sanskrit, Pāli, Apabhraṃśa, and Old Avadhī,1There is no direct continuity between these languages, which are merely examples of subsequent stages in the history of the Indo-Aryan languages. Yet, for the study of case syntax, this is not a major problem, given that “the development of postpositions and postpositional phrases is a systematic one that is found in all the New Indo-Aryan languages spoken in South Asia” (Reinöhl 2016a: 11).
Reinöhl (2016a, 2016b) has, for instance, been able to trace the grammaticalisation process that resulted in two postpositional case markers found in modern Hindi: (i) *mẽ* ‘in’ < OIA *madhye*, loc.sg of *madhya*- ‘middle’, and (ii) *par* ‘on’ < OIA *upari* ‘above, on top’.2For the etymologies, see Reinöhl (2016a: 54–57). Interestingly, the Iranian cognate of *upari* ‘above, on top’ also became grammaticalised as a superessive case in Ossetic (Belyaev 2010: 300–301). Contrary to a common assumption found in the literature (e.g. in Bubeník 2006), Reinöhl argues forcefully against a direct continuity between such case markers and the Vedic local adverbs like *ádhi* ‘above’ (Reinöhl 2016a: 65–83, 2016b). As shown by her, the major source for the NIA postpositional cases are rather “relational noun constructions with a possessor encoded by the genitive”, i.e. constructions such as “in the middle of X” or “on the top of X”. As part of her argument, Reinöhl identifies three grammaticalisation processes: (i) the relational noun construction gradually loses its originally more concrete semantics and comes to be reinterpreted as a postposition encoding grammatical case relations (2016a: 85–117, 2016b: 111–116); (ii) there is a development from free word order to a fixation of the order genitive + postposition, although even in Apabhraṃśa this has not yet become a compulsory rule (2016a: 119–145, 2016b: 116–121); and (iii) a genitive complement is in origin not obligatory with these relational noun constructions but becomes so over time (2016a: 147–173, 2016b: 121–123). Finally, Reinöhl observes that these genitive complements tend to be animate nouns and pronouns, although this preference for animates is not (yet) a fixed rule in the MIA languages studied by her (2016a: 158–162).3For Pāli, the preference for animate genitive complements had been noted before; cf. e.g. Fahs (1985: §163.4, p. 105).


While Reinöhl’s studies certainly are “a bench mark for further studies on Indo-Aryan postpositions” (Verbeke 2018: 123), a drawback to her work is that she did not include data from non-literary sources such as Prakrit inscriptions or remarks by ancient grammarians (Verbeke 2018: 123–124). This exclusive reliance on literary texts stands in contrast to recent work on the development of case marking in both Greek and Arabic, where the importance of a detailed linguistic study of documentary texts written on papyrus is rightly emphasised (cf. e.g. Stolk 2017 for Greek and Kootstra 2022 for Arabic).

The only text corpus in a MIA language that can be compared in content and form to documentary papyri are the so-called Niya documents (3rd–4th century CE), which were drafted in a dialect of Central Asian Gāndhārī known as Niya Prakrit.4For a grammar of Niya Prakrit, see Burrow (1937) and for the phonological interpretation now also Schoubben (2024: 17–40). In what follows, quotations from the Niya documents are taken from the online “Catalog of Kharoṣṭhī Documents” (CKD) edited by Baums and Glass (2002–); but, in contrast to Baums and Glass, I transliterate the Kharoṣṭhī sign 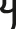 as *jh* and not as *z*, for the reasons given in Schoubben (2024: 29–36). As I will argue in Schoubben (in prep.), I further assume that the following Niya documents can be joined together: CKD 195 + 726, CKD 222 + 336, CKD 271 + 338, and CKD 324 + 328. In the present paper, I generally restrict myself to documents written in Niya Prakrit, leaving aside the other forms of Central Asian Gāndhārī, i.e. Khotan Prakrit (CKD 661; 843) and Kuča Prakrit (CKD 829–838; 844–845; 870–877; 883; 891–896). See, however, Section 6 for some notes on a Khotan Prakrit postposition. In this paper, I would therefore like to take a closer look at the case system of this MIA language, in particular its ablative and locative.5As we shall see in Section 4, the locative case can also be used as an allative or (more rarely) a dative. Drawing on Reinöhl’s observations on the role played by animacy in the selection of synthetic versus analytic case marking in Indo-Aryan languages (see above), I aim to demonstrate that (i) the Niya Prakrit synthetic ablative and locative are basically restricted to inanimate nouns, and that (ii) the postposition *paride* ‘from’ supplies an analytically formed ablative for animate nouns and pronouns, while *vaṃti* ‘in, at, to’ does the same for the locative. The approach will be mainly synchronic and language-internal, but occasional comparisons with both Indo-Aryan and non-Indo-Aryan languages will allow us to frame the findings of this article within a diachronic and typological perspective.

The article consists of the following parts: a brief introduction to the Niya Prakrit case system (Section 2); an investigation into the postposition *paride* ‘from’ and the synthetic ablative (Section 3); a parallel investigation into the postposition *vaṃti* ‘in, at, to’ and the synthetic locative (Section 4); a discussion of the Niya Prakrit personal pronouns from the perspective of animacy (Section 5); a note on the Khotan Prakrit postposition *sag̲aj̲i* ‘in, at, to’ (Section 6); and some concluding remarks (Section 7). The data on which the findings of this article are based can be found in the Appendix.

## 2 A brief introduction to the Niya Prakrit case system

To facilitate the discussion on the ablative and locative below, I briefly summarise here the main points about the Niya Prakrit case system (Burrow 1937: §§51–73, pp. 22–30).

In line with other MIA languages, the Niya Prakrit declensional system has been greatly simplified when compared to the rich morphology of OIA. As also happened in Apabhraṃśa, nominative and accusative have been merged in Niya Prakrit to what I shall call the “direct case” (dir) (following Baums 2009: 211 *et passim*).6Third person pronouns still distinguish between nominative and accusative, which makes Niya Prakrit a “case-asymmetrical language” (Iggesen 2009), comparable to, e.g., English, where pronouns distinguish direct versus oblique, but nominals do not. The fact that the asymmetry is limited to third person pronouns makes Niya Prakrit belong to a typologically less frequent subtype of case-asymmetrical languages (cf. Iggesen 2009: 251). While there are traces left of other declensions (Burrow 1937: §§68–72, pp. 27–29), only the *a*-declension still enjoys large productivity in Niya Prakrit. The paradigm of this declension is exemplified below, using the word *bhuma*- ‘land’ as a model; not all forms given are actually attested for the word *bhuma*-, and infrequent variant endings are indicated in between brackets.

**Table:** 

	sg	pl
dir	*bhum-a*	*bhum-a* (-*e*)
ins	*bhum-ena*	*bhum-ehi*
gen	*bhum-as̲a* (-*asya*)	*bhum-ana* (-*anaṃ*)
abl	*bhum-ade* (-*āde*)	–
loc	*bhum-a(ṃ)mi* (-*e*)	*bhum-eṣu*

The abl.sg ends in -*ade* (-*āde*), where the occasional spelling with a long vowel is probably to be taken seriously given the exact cognates in Śaurasenī -*ādo* and Mahārāṣṭrī -*āo*;7The long vowel in this MIA ending, which differs from the OIA ending in -*ataḥ*, is usually explained as resulting from a blend between -*ataḥ* and the OIA abl.sg in -*āt* (thus, e.g., von Hinüber 2001 [1986]: §302, pp. 224–225). no abl.pl is attested so far, though one would expect it to take the same form as the ins.pl, i.e. -*ehi* (cf. Burrow 1937: §63; p. 26). The loc.sg in -*a(ṃ)mi* derives from the OIA pronominal ending -*asmin*; the old nominal ending in -*e* is only rarely attested in Niya Prakrit, and the same holds for the loc.pl in -*eṣu* (cf. Section 4.2).

## 3 The postposition *paride* ‘from’ and the synthetic ablative

In this section, I aim to show that there is a complementary distribution between the Niya Prakrit postposition *paride* and the synthetic ablative: they are used in the same function, viz. to indicate a source; but the former is used with animate nouns, while the latter is restricted to inanimates. Since the postposition *paride* ‘from’ has received but scanty attention in the literature (Burrow 1937: 41), I will in addition elaborate on its semantics and etymology.

### 3.1 The postposition *paride* ‘from’

Etymologically, Niya Pkt. *paride* ‘from’ must be compared to OIA *paritaḥ* ‘around’ (Turner 1966–1985 I: 442 no. 7830). Yet, the semantic difference between these two forms deserves a few comments. In my view, the difference has to do with a morphosyntactic distinction between *paride* and *paritaḥ*: whereas the latter is used as a preposition governing an accusative,8I know of one exceptional case where *paritaḥ* governs a genitive, i.e. Rām. 2.81.22cd *mahad dhanuḥ sajyam upohya lakṣmaṇo niśām atiṣṭhat* ***parito ’sya*** *kevalām* (v.l. *kevalam*) “Holding his big bow strung, Lakṣmaṇa stood **around him** during the entire night.” (tr. mine; *parito* = sandhi form of *paritas* ‘around’; *’sya* = gen.sg *asya* ‘he’). the former is a postposition constructed with a complement in the genitive. As a result, Niya Pkt. *paride* is probably to be understood as an example of the “post-Vedic genitive shift”: the reanalysis of adjectival and adverbial elements as relational noun expressions taking a genitive (Reinöhl 2016a: 94–110, 2016b: 113–116). A well-known example of this post-Vedic genitive shift is the spatial adverb *upari* ‘above, on top’, which can take a genitive complement in later forms of Indo-Aryan – but not yet in Vedic. Probably, *upari* came to be reanalysed as a loc.sg in -*i* of a consonantal stem, allowing it to take a genitive complement in front (thus Bloch 1965 [1934]: 179; Reinöhl 2016a: 100). Applying the same reasoning to *paride*, this originally adverbial form can have been reanalysed as an abl.sg in -*de* (< OIA -*taḥ*) to a nominalised element *pari* ‘around > entourage’, with a straightforward semantic development of ‘from around; out of the entourage of’ to ‘from’.9One should probably resist the temptation to find a direct link between Niya Pkt. *paride* ‘from’ and the use of ablative + *pári* ‘around’ meaning ‘from X’ in the *R̥gveda*. As Hettrich (2002: 233–236) has shown, this Vedic construction merely shows a desemanticisation of *pári*, as it is the ablative and not *pári* which encodes the meaning ‘from’ (cf. also Bubeník 2006: 109–110). Such a reanalysis could have been facilitated by a perceived parallelism with the locative postposition *vaṃti* ‘in, at, to’, which is derived from the relational noun *upānte* (loc.sg) ‘in the proximity of > in, at, to’ (see Section 4.1). One can further compare examples such as Pāli gen + *pacchato* ‘behind’ versus Vedic *paścā́* ‘idem’, for which Reinöhl (2016a: 97) argues that “the added case suffixes [here -*to* < -*taḥ*] lend these elements a nominal form and a slot for genitives along with it”.

Basing myself on all the occurrences of *paride* in the Niya Prakrit corpus (∼149 usable tokens; see Appendix 1), I have investigated the degree to which *paride* has been grammaticalised. For this, I looked at the three factors identified by Reinöhl (2016a: 85–173) in her examination of the outcomes of OIA *madhye* ‘in the middle of’ and *upari* ‘above, on top’ (cf. Section 1). Applied to *paride,* they are: (i) are there traces left of the original semantics, or has *paride* been fully reanalysed as a case marking postposition; (ii) is the order gen + *paride* obligatory, or is the opposite order, i.e. *paride* + gen, possible too; and (iii) is the genitive complement compulsory, or is *paride* also found on its own? The noteworthy result of this examination is the sheer consistency which *paride* shows regarding all three factors: (i) *paride* by default encodes an ablatival relationship, indicating an, as we shall see, animate source; (ii) the genitive complement always stands in front of *paride* and is directly adjacent to it; and (iii) *paride* never occurs without a complement in the genitive.10There are three examples where the genitive ending is missing in front of *paride*: *toṃga Suḡ̱iya paride* ‘from the *toṃga* [an official title] Suḡ̱iya’ (CKD 208 o1); *Vug̱eyas̱a putra paride* ‘from the son of *Vug̱eya*’ (CKD 546 o2–3); *Kapg̱eya paride* ‘from Kapg̱eya’ (CKD 568 co1). These examples could be seen as the next stage in the development of *paride*, whereby the postposition was becoming a cliticised case marker (cf. Bubeník 2006: 119 on a similar development in later Apabhraṃśa), but one can alternatively assume scribal omissions of the akṣara ⟨s̲a⟩. Interestingly, Reinöhl, in her study of Indo-Aryan postpositions, observed a similar consistency only in 16th-century texts in Old Avadhī (more on this in Section 7).

The ensuing sentences are meant to exemplify the most characteristic usages of *paride* in Niya Prakrit. In (1), the source of information is indicated by *tahi paride* ‘from you’, while the content of what is heard is expressed by the dir.sg *arog̱a* ‘health’.

(1)

**Table:** 

*tena*	*ṣademi*	***tahi***	***paride***	*arog̱a*
this.ins.sg	be.pleased.prs.1sg	**you.** **gen** **/** **dat** **.** **sg**	**from**	health.dir.sg
*śrudemi*				
hear.pst.1sg				
‘I am pleased because I have heard **from you** that you are in good health.’				
(CKD 139 o3–4; tr. Burrow 1940: 25)				

Another representative example comes from CKD 345, here given as (2). Freely paraphrased, this sentence says that corn was transferred at a certain point of time from Cug̱opa (*Cug̱opas̱a paride*), i.e. the source, and was subsequently received by the monk Anaṃdas̱ena (*śramaṃna Anaṃdas̱ena*).

(2)

**Table:** 

*bhudartha*	*Caḍ̱otaṃmi*	*śramaṃna*	*Anaṃdas̱ena*	***Cug̱opas̱a***
truly	Caḍ̱ota.loc.sg	monk.dir.sg	Anaṃdas̱ena.dir.sg	**Cug̱opa.** **gen** **.** **sg**
***paride***	*aṃna*	*av̱amicae*	*giḍ̱aka*	*huati*
**from**	corn.dir.sg	on loan	receive.pst.ptcp	be.prs.3sg
‘It is a fact that in Caḍ̱ota the monk Anaṃdas̱ena received … corn on loan from Cug̲opa [PN].’				
(CKD 345 uo2; tr. Burrow 1940: 65)				

In a few cases, as e.g. in (3), one could be tempted to interpret a noun phrase governed by *paride* instead as the agent of a patient-oriented sentence, comparable to the usage of *hačā ‘from’ and its derivatives as a marker of agency in various Iranian languages.

(3)

**Table:** 

***Apig̱oas̱a***	***paride***	*parikraya*	*iśa*	*Tuṣanaas̱a*	*prahatavo*
**Apig̱o.** **gen** **.** **sg**	**from**	wages.dir.sg	here	Tuṣana.gen.sg	send.ger
‘… wages are to be sent here **from Apig̱o [PN]** to Tuṣana.’					
(CKD 30 o3–4; tr. Burrow 1940: 7)					

Yet, it seems better to resist such an interpretation, as there are a few examples where in one and the same sentence an agent in the genitive case is found next to a noun phrase with *paride* ‘from’.11See e.g. CKD 39 cr1: *tirsa vaḍ̱avi atha va tirṣa aśpa* **[*Kapg̱eyas̱a dajhana paride*]**_**source**_ **[*Lýipeyas̱a*]**_**agent**_ *nidavo* “(If so) a *tirsa* mare or a *tirṣa* horse is to be taken **[by Lýipeya]**_**agent**_ **[from the slaves of Kapg̱e]**_**source**_” (tr. Burrow 1940: 9–10). In CKD 633 r6–7, it seems possible to interpret *paride* ‘from’ as a marker of agency, but this remains an isolated example: *eda kalaśa 2* **[*Priyavatas̱a paride*]**_**agent?**_ *śodhedavo* “These two jars are to be paid off **[by Priyavata]**_**agent?**_.” (tr. Burrow 1940: 131). In the case of (3), we are simply informed that the wages are transported from their original location at Apig̱o’s place, not necessarily that it is Apig̱o who sends them to Tuṣana.

In addition, there are some examples where noun phrases with *paride* ‘from’ are, at first sight at least, not the complement of a verb but constructed adnominally so as to resemble a possessive genitive.12Such a development of “from X” to a possessive marker is also typologically common; cf. Heine (2009: 464–465). An example is provided in (4).

(4)

**Table:** 

*eṣa*	*paṭi*	***Suḡ̱iyas̱a***	***paride***	*kuḍ̲i*	
this.nom.sg	tablet.dir.sg	**Suḡ̱i.** **gen.sg**	**from**	girl.dir.sg	
*Sag̱anāpaae*	*pra[ce]*	*Maṣḍhig̱eyas̱a*	*anada*		
Sag̱anāpaae.dir.sg	concerning	Maṣḍhig̱e.gen.sg	carefully		
*dharidavo*					
hold.ger					
‘This tablet (*paṭi*) concerning a girl Sag̱anāpaae **(bought) from Suḡ̱i [PN]** is to be carefully preserved by Maṣḍhig̱e.’					
(CKD 437 co1–2; tr. Burrow 1940: 89)					

While one could be inclined to interpret *Suḡ̱iyas̱a paride* ‘from Suḡ̱i’ in (4) as adnominal with *kuḍ̲i* ‘girl’, approximately meaning ‘Suḡ̱i’s girl’, such an interpretation is shown to be unlikely by the rest of CKD 437: Suḡ̱i has sold the girl and thus is no longer her owner, which means that, as Burrow does in his translation, one should assume an unexpressed participle dir.f.sg *kriti ‘bought’ in between *Suḡ̱iyas̱a paride* ‘from Suḡ̱i’ and *kuḍ̲i* ‘girl’.

The examples (1) to (4) all have in common that the complement of *paride* is an animate noun or pronoun. In itself, this is not unexpected: (i) Fahs (1985: §163.4, p. 105) and Reinöhl (2016a: 158–162) have previously observed a preference for animate complements with postpositions in Pāli and Apabhraṃśa (cf. Section 1); (ii) animacy plays a crucial role in the case marking of various NIA languages (see e.g. Lahiri 2021 on Eastern NIA); and (iii) it is cross-linguistically common that nouns referring to animate beings use special forms involving adpositions to express spatial case relations (cf. e.g. Creissels 2009: 612–613; Santazilia 2023: 63; 136–137). But it is still remarkable that *paride* ‘from’ is *always* constructed with personal names, nouns referring to animate entities,13Animals such as “horses” or “camels” also count as animate nouns in Niya Prakrit; cf. e.g. *uṭiyana paride* ‘from the she-camels’ in CKD 125 o1. With the limited data at hand, it is difficult to say whether all animals are treated in this way, as they, e.g., are in eastern NIA languages (cf. Lahiri 2021: 177), or that some animals would be treated as animate and others (e.g. insects) as inanimate. For this last option, cf. de Swart, Lamers and Lestrade (2008: 131; 135 with further ref.), who also point out that some languages treat all animals as inanimates. and animate pronouns, whereas in Pāli and Apabhraṃśa, there is only a *tendency* for postpositions to combine with animates.

### 3.2 The synthetic ablative

Now that it is clear that the postposition *paride* ‘from’ is restricted to animate nouns, a new question poses itself: is the synthetic ablative then confined to inanimates? To address this question, I have collected all the Niya Prakrit abl.sg in -*ade* (-*āde*) with the help of the digital text corpus (Baums and Glass 2002–).14As noted in Section 2, there are no abl.pl attested in Niya Prakrit. Leaving pronouns aside (for which see Section 5), I counted 77 types, 273 tokens of synthetic ablatives (listed in Appendix 2b). And, importantly, all but one of these are inanimates: in particular nouns referring to locations (e.g. toponyms like *Caḍ̱otade*) and expressions of time (e.g. *varṣade* ‘year’).15I have counted *senade* ‘from the army’ (CKD 399 o5) / *seniyade* ‘idem’ (CKD 562 uo2) also as an inanimate noun contrastive with *seni jaṃnana paride* ‘from the army people’ (CKD 291 o5). I would further argue that in the case of the word *Supi* (also *Supiya*), we have to distinguish between two homonyms, one referring to the place “Supi” and one to the people living there, the “Supis”. Then, the abl.sg *Supiyade* (CKD 515 uo3; 722 uo6) can be translated as ‘from Supi [toponym]’, which is supported by the collocation of *Supiyade* with the abl.sg *Calmadanade* ‘from Calmadana [toponym]’ in CKD 722 uo6.


The only clearly animate noun inflected in the ablative is the PN *Apg̱eyade*; see (5).

(5)

**Table:** 

*eda*	*prace*	*tu*	***Apg̱eyade***	*anati*
this.acc.sg	concerning	you.dir.sg	**Apg̱eya.** **abl.sg**	command.dir.sg
*giḍ̱esi*				
receive.pst.2sg				
‘About this matter you received a command **from Apg̱eya [PN]**.’				
(CKD 63 uo2–3; tr. Burrow 1940: 14)				

The two other instances of *anati* (*anadi*) *giḍ̱esi* ‘you received a command’ in the Niya Prakrit corpus (CKD 63 uo4; 144 uo3) are constructed with the inanimate abl.sg *asiyade* ‘from the mouth (of)’, together meaning ‘you received an oral command (from person X)’. Therefore, I tentatively suggest that the anomalous *Apg̱eyade* is a *saut du même au même* for *Apg̱eya*⟨*s̱a asiya⟩*de* ‘from the mouth of Apg̱eya’, whereby the scribe inadvertently mistook the ⟨ya⟩ of *Apg̱eya*⟨*s̱a⟩ as that of ⟨*asiya⟩*de*. *Apg̱eyade* would not be the only scribal mistake in the sentence, as the dir.sg *tu* ‘you’ right in front of it is probably a misspelling for *tu*⟨*o⟩ ‘you’ (thus already Burrow 1937: §79, p. 32).

Be that as it may, I shall now provide further evidence that (i) the postposition *paride* ‘from’ and the synthetic ablative fulfil the same syntactic function in similar contexts, and that (ii) the two constructions are distinguished by animacy. This evidence takes the form of pairs of example sentences whereby the same verb is used in both instances, and whereby the synthetic ablative indicates an inanimate source and the postposition *paride* an animate source.

In (6a) and (6b), the verb used is *de(na)*- ‘to give’ (Skt. √*dā*).16For further examples with this verb, see CKD 583 uo3–4; 630 o3–4 (with *paride*) and CKD 14 uo3, cr1; 55 uo2; 64 o3 (2×); 214 o3–4; 223 uo2, uo3 (2×); 272 o7; 296 uo2; 306 r1; 358 o6, o7; 367 o2, o3; 502 o2–3, o4 (with the synthetic abl).

(6a)

**Table:** 

*cavala*	***dadavo***	*yo*	*laṃcag̱a*	*siyati*	***tava***
quickly	**give.** **ger**	which	right.dir.sg	be.opt.3sg	**you.** **gen./dat.sg**
***paride***					
**from**					
‘Quickly what is right **must be given** him **from you**.’					
(CKD 161 uo5–6; tr. Burrow 1940: 30)

(6b)

**Table:** 

***Calmadanade***	*valag̱a*	***ditaṃti***
**Calmadana.** **abl.sg**	guard.dir.sg	**give.pst.3pl**
‘**From Calmadana [toponym] they gave** him a guard.’		
(CKD 14 uo2–3; tr. Burrow 1940: 3)		
(7a) and (7b) provide examples with the verb *palay*- ‘to flee’ (Skt. √*palāy*).^17^		

17 (7a) is the only example where *palay*- is combined with *paride*. For more examples of its being combined with the synthetic abl, see CKD 216 r1; 532 uo4; 540 uo3; 621 o3; 842 o3, o4, o8, o9.

(7a)

**Table:** 

*se*	*palayaṃnag̱a*	***Jeyakas̱a***	***paride***	*puna*
this.nom.sg	refugee.dir.sg	**Jeyaka.** **gen.sg**	**from**	again
***palayita***				
**flee.pst.3sg**				
‘That refugee **fled** again **from Jeyaka [PN]**.’				
(CKD 403 uo3; tr. Burrow 1940: 83)

(7b)

**Table:** 

*se*	*Sag̱amovi*	*stri*	*Supriyae*	*s̱adha* 18
this.nom.sg	Sag̱amovi.dir.sg	woman.dir.sg	Supriya.ins.sg	with
***Caḍ̲otade*** 19	***palayitaṃti***			
**Caḍ̲ota.** **abl.sg**	**flee.pst.3pl**			
‘This Sag̱amovi and his wife Supriya **fled from Caḍ̲ota [toponym]**.’				
(CKD 884 uo6–7; tr. mine)				

18 In Duan (2016: 57), this word is read as *s̱aca*, but the accompanying picture shows that we should read *s̱adha*, just like in CKD 884 uo6.

19 My reading; Duan (2016: 57) has *Caḍotade*.

In (8a) and (8b), we have examples with the verb *preṣ-* ‘to send’ (Skt. *pra* + √*iṣ*).20For *preṣ-* ‘to send’ + *paride* ‘from’, see further CKD 288 cr3–4 and CKD 431 cr2 = 432 uo7 (with prs.1sg *eṣemi* ‘I send’); and for *preṣ-* ‘to send’ + synthetic abl CKD 320 r7.

(8a)

**Table:** 

*lahu*	*manas̱ikara-matra*	*prahuḍ̲a*	
small.dir.sg	but.a.token.of.remembrance.dir.sg	present.dir.sg	
***preṣitama***	***Kuktae***	***paride***	*chotag̱a*
**send.pst.1pl**	**Kukta.** **gen.sg**	**from**	*chotag̱a*.dir.sg
***Parpanas̱a***	***paride***	*lastug̱a*	
**Parpana.** **gen.sg**	**from**	*lastug̱a*.dir.sg	
‘**We have sent** a small present as a token of thoughtfulness, **from Kukta [PN]** one *chotag̱a* [an unidentified object], **from Parpana [PN]** one *lastug̱a* [a textile product].’^21^			
(CKD 161 cr5–6; tr. Burrow 1940: 30)			

21 We know that Kukta (variant spelling Kukita) and Parpana are personal names because they are the authors of the letter from which the sentence above has been taken (see uo2–3).

(8b)

**Table:** 

***Puṣg̱ariyāde***	*rayag̱a*	*paṭa*	***preṣitaṃti***
**Puṣg̱ari.** **abl.sg**	royal.dir.sg	silk.roll.dir.sg	**send.pst.1pl**
**‘** **From Puṣg̱ari [toponym] they sent** 1 roll of royal silk.’^22^			
(CKD 660 oA2; tr. Burrow 1940: 136)			

22 For the idea that Puṣg̱ari is a toponym, cf. Puṣg̱a[ri](*yaṃmi) paryaṃta ‘as far as Puṣg̱ari’ in CKD 770 uo4 (Schoubben 2024: 384). Note further that the abl.sg *khvaniyāde* ‘from the capital city’ is used in the same function as *Puṣg̱ariyāde* in the preceding line (CKD 660 oA1).

Particularly interesting are examples such as (9) and (10), where in the same sentence the synthetic ablative is used for an inanimate complement and *paride* for an animate one; in (9), the verb is *ciṃd*- ‘to assess’ (Skt. √*cint*), and in (10), it is *pruch*- ‘to ask’ (Skt. √*prach*).

(9)

**Table:** 

*yo*	***kilmeciyana***	***paride***	*yaṃ*	*ca*
which	**belonging.to.a.family.estate.** **gen.pl**	**from**	which	and
***rajade***	*palýi*	***ciṃditag̱a***		
**state.** **abl.sg**	tax.dir.sg	**assess.** **pst.ptcp**		
‘… which tax **was assessed from the people on a family estate** and which (tax **was assessed) from the state**.’				
(CKD 374 o2; tr. mine)

(10)

**Table:** 

***tade***	***bhumade***	*nevi*	*kori*	*Muldeyas̱a*
**this.** **abl.sg**	**land.** **abl.sg**	neither	animal.supervisor.dir.sg	Muldeya.gen.sg
***Ramṣotsas̱a***	***paride***	*vag̱a*	*aṃna*	***pruchidavo***
**Ramṣotsa.** **gen.sg**	**from**	rent.dir.sg	corn.dir.sg	**demand.** **ger**
*nevi*	*…*			
nor	…			
**‘From that land** neither is corn as rent **to be demanded of Ramṣotsa [PN]** by the animal supervisor (*kori*) Muldeya nor …’				
(CKD 574 cr1–2; tr. after Burrow 1940: 116)				

## 4 The postposition *vaṃti* ‘in, at, to’ and the synthetic locative

In this section, it will be shown that animacy does not only affect the marking of ablatival relationships in Niya Prakrit. The synthetic locative is restricted to inanimates as well, whereas for animate nouns, locatival relationships are expressed with the postposition *vaṃti* ‘in, at, to’.

### 4.1 The postposition *vaṃti* ‘in, at, to’

The Niya Prakrit postposition *vaṃti* ‘in, at, to’ is etymologically derived from the OIA relational noun *upānte* ‘in the proximity of’, loc.sg of *upānta*- (cf. Burrow 1937: §92, 42–43).23For the raising of final -*e* to -*i* in *vaṃti*, see Burrow (1937: §1, p.1). Burrow’s idea (1937: §92, 42–43) that the Khotanese postposition *bendä* ‘on’ + gen/dat is a borrowing from Niya Prakrit *vaṃti* must probably be rejected because of the Pashto cognate *bā́nde* ‘on, upon, above’ (cf. Konow 1938: 155; Skjærvø 2004 II: 320). One may further observe that *bendä*, in contrast to *vaṃti* (cf. infra), often governs inanimate nouns, e.g. *āysanānu bendä* ‘on seats’ as a translation of the Skt. loc.pl *āsaneṣu* ‘idem’ (Sgh 70.3; 70.4; Canevascini 1993: 31); and that Khotan Prakrit, the dialect of Gāndhārī used in the Khotanese-speaking area, may not have used the postposition *vaṃti* but the synonymous *sag̲aj̲i* (see Section 6). In structure and meaning, *vaṃti* can be compared to Pāli postpositions such as *santike* ‘in the presence of > with, before’, loc.sg of *santika*- ‘vicinity, presence’ or *samīpe* ‘in the proximity of > near’, loc.sg of *samīpa*- ‘proximity’ (Fahs 1985: §165, p. 110).

Basing myself on all the instances of *vaṃti* ‘in, at, to’ in the Niya Prakrit corpus (∼187 usable tokens; see Appendix 3), I have examined the same three parameters as for *paride* ‘from’ (cf. Section 3.1): (i) the semantics of the postposition; (ii) the relative order of the genitive complement and the postposition; and (iii) the obligatoriness of the genitive complement. The results of this examination are in line with those of *paride*: (i) semantically, *vaṃti* no longer preserves unambiguous traces of its original meaning ‘in the proximity of’ but encodes locatival functions (which in Niya Prakrit include “allative” and “dative”); (ii) the genitive complement is always put in front of *vaṃti*; and (iii) the genitive complement is obligatory.24In one instance, i.e. *śramaṃna Anaṃdas̱ena vaṃti* (CKD 345 uo12), the gen ending -*s̲a* (or -*sya*) is missing, presumably because of a scribal omission (cf. fn. 10). In 184 instances (∼98.4 %), the genitive complement is also an animate noun.25Only three counterexamples exist (∼1.6 %), all of which are moreover of the same type, being complements of *eśvarya huda* ‘he had possession of’: CKD 579 uo5: *edas̱a bhumas̱a vaṃti* ‘in this piece of land’; 580 uo7: *akriya bhumas̱a vaṃti* ‘in *akri* land’; 582 uo4: *tas̱a bhumas̱a vaṃti* ‘in this piece of land’. See also fn. 37. Thus, like *paride*, *vaṃti* by default selects animate complements.

In what follows, I shall briefly exemplify the morphosyntactic usage of *vaṃti*, starting with an example of *vaṃti* in a purely locatival meaning (11).

(11)

**Table:** 

*Budhas̱ena*	*viṃñaveti*	*yatha*	*edas̱a*	*uṭa*	*1*
Budhas̱ena.dir.sg	inform.prs.3sg	that	this.gen.sg	camel.dir.sg	1
***Kolýisas̱a***	***vaṃti***	*huati*			
**Kolýisa.** **gen.sg**	**in, at, to**	be.prs.3sg			
‘Budhas̱ena informs us that he had a camel **with Kolýisa [PN]**.’					
(CKD 356 o2; tr. Burrow 1940: 69)					

Additionally, *vaṃti* can function as an allative or a dative, and it is often difficult to distinguish between these two functions; so also in (12) and (13).

(12)

**Table:** 

*bahu*	*cirakala*	*huda*	*na*	*śakidama*	
many.dir.sg	a.long.time.dir.sg	be.pst.3sg	neg	be.able.pst.1pl	
***tehi***	***vaṃti***	*lekha*	*prahuḍ̱a*	*preṣaṃnae*	
**you.** **gen./dat.sg**	**in, at, to**	letter.dir.sg	present.dir.sg	send.inf	
‘It is a long time since we were able to send **you** a letter and a present.’					
(CKD 288 uo3–4; tr. Burrow 1940: 52)

(13)

**Table:** 

*Argiceyas̱a*	*bhratarana*	***Kuv̱ayas̱a***	***vaṃti***
Argiceya.gen.sg	brother.gen.pl	**Kuv̱aya.** **gen.sg**	**in, at, to**
*buma*	*vikridati*		
land.dir.sg	sell.pst.3pl		
‘Argiceya and his brothers sold land **to Kuv̱aya [PN]**.’			
(CKD 422 o4; tr. Burrow 1940: 86)			

To determine the syntactic function of *tehi vaṃti* ‘to you’ (12) and *Kuv̱ayas̱a vaṃti* ‘to Kuv̱aya’ (13), one must decide whether the emphasis lies on receiving the letter/present (*lekha*; *prahuḍ̱a*) and the piece of land (*buma*) or whether it is rather the endpoint of movement that is highlighted. In the first case, *tehi vaṃti* and *Kuv̱ayas̱a vaṃti* could be seen as dative complements indicating recipients, while in the second case, they may rather be allatives indicating a goal. But, irrespective of which option one prefers,26Given that the present/letter are literally sent towards another person, while the piece of land is bought and thus not physically changing place, I would interpret *tehi vaṃti* ‘to you’ as indicating a goal and *Kuv̱ayas̱a vaṃti* ‘to Kuv̱aya’ as a recipient, but it is a fine distinction. one can see that the postposition *vaṃti* exemplifies the typologically frequent polysemy between allative (goal) and dative (recipient) (Malchukov and Narrog 2009: 520).27In Niya Prakrit (and other MIA languages), recipients are normally marked in the genitive case. It should therefore be investigated further in which contexts the postposition *vaṃti* is preferred and in which a plain genitive. Pending such an investigation, I would briefly want to draw here a parallel between Niya Pkt. *vaṃti* and the Latin preposition *ad* ‘in, at, to’. Originally mainly encoding spatial relationships, the outcomes of *ad* in various Romance languages, including French *à*, came to be used as a dative marker. Although it has often been asserted that the Romance situation is already prefigured in Late and Vulgar Latin, Adams (2013: 278–294) was able to show that *ad* is even in late texts not fully interchangeable with the dative case, and that the usage of *ad* often implies motion and travel over a distance. It is my impression that the same holds true for *vaṃti,* but this hypothesis needs testing, as said before, on the basis of a more comprehensive investigation of the distribution between *vaṃti* and the use of the genitive for recipients.


The usage of *vaṃti* parallels that of the synthetic locative case, whose functions likewise range from a locative proper over an allative to occasionally even a dative. In (14), for instance, the loc.sg *pirovami* ‘fortress’ is prototypically locatival in that it indicates the place where a certain sacrifice (*goyaṃña*) happened.

(14)

**Table:** 

*avi*	*ca*	***pirovami***	*Bhatro* 28	*devatas̱a*
also	and	**fortress.** **loc.sg**	Bhatro.dir.sg	divinity.gen.sg
*goyaṃña*	*huda*			
cow-sacrifice.dir.sg	be.pst.3sg			
‘Also there has been a sacrifice of a cow **at the fortress** to the god Bhatro (Tsatro?).’				
(CKD 157 o3; tr. after Burrow 1940: 28–29)				

28 ⟨bh⟩ and ⟨ts⟩ are not well-distinguished in the type of Kharoṣṭhī script used to write Niya Prakrit, so this name could also be read as *Tsatro*.

In example (15), the loc.sg *Khotaṃnaṃmi* ‘Khotan’ indicates the direction to which the *ogu* [an official title] Alýaya is sent on an embassy and is thus a locative used in the function of an allative (cf. Burrow 1937: §123, p. 60).29Similarly, *iśa* (∼ OIA *iha*) means not only ‘here’ in Niya Prakrit, but also ‘hither’.

(15)

**Table:** 

ahun(*o)	*ogu*	*Alýayena*	***Khotaṃnaṃmi***
now	*ogu*.dir.sg	Alýaya.ins.sg	**Khotan.** **loc.sg**
*dutiyae vis̱ajidemi*			
to.send.on.a.mission.pst.1sg			
‘Now I have sent the *ogu* [an official title] Alýaya on a mission **to Khotan**.’			
(CKD 214 o2; tr. Burrow 1940: 40)			

Rarely, the locative is even used to indicate a recipient in Niya Prakrit, a function typically associated with dative cases.30The restriction of the synthetic locative to inanimates (see Section 4.2) accounts for the rarity of this datival function, as recipients are by definition animates rather than inanimates. Compare example (16), where the loc.sg *Petaav̱anaṃmi* ‘in Peta-village’ indicates the recipient of *didemi* ‘I gave’.

(16)

**Table:** 

*ahuno*	*ahaṃ*	*maharaya*	***Petaav̱anaṃmi***
now	I.dir.sg	great.king.dir.sg	**Peta-village.** **loc.sg**
*palayaṃnag̱a*	*didemi*		
refugee.dir.sg	give.pst.1sg		
‘Now I the great king have handed over [literally: “given”] a fugitive man **to Peta-village**.’			
(CKD 136 uo1–2; tr. after Burrow 1940: 24)			

### 4.2 The synthetic locative

The next question is whether synthetic locatives are, like the synthetic ablatives (Section 3.2), restricted to inanimate nouns. To address this, I first collected all the instances of the loc.sg in -*a(ṃ)mi* 31The unproductive loc.sg in -*e* has been left aside. in CKD 1–400, i.e. in somewhat less than half of the currently available Niya documents. The data thus collected amounts to 85 types, 545 tokens; see Appendix 4a.32I restricted my search to CKD 1–400 because locatives are considerably more frequent than ablatives. Even with this restriction, we have more data for the locative than for the ablative, so that it is unlikely that an examination of the remaining Niya documents would significantly alter the picture presented below. Notably, nearly all of these loc.sg are inanimate nouns, e.g. indications of time like *varṣaṃmi* ‘year’; only one is an animate noun, viz. loc.sg *maṃnuśami* ‘man’ in CKD 324 + 328 cr1 (see just below).

Secondly, I counted 17 types, 25 tokens of the loc.pl in -*eṣu*; see Appendix 4b. With these, too, a clear preference for inanimates can be noted, as 13 types (∼76.47 %), 21 tokens (∼84 %) are inanimates. Yet, there are some more counterexamples than for the singular nouns: *uṭiyeṣu* ‘she-camels’ (CKD 134 o2); *Calmatāneṣu* ‘the people of Calmadana’ (CKD 119 o4); *paśuveṣu* ‘sheep’ (CKD 568 uo4–5); and *Sāceṣu* ‘the people of Saca’ (CKD 637 o10); i.e., 4 types (∼23.53 %), 4 tokens (∼16 %).33As noted in fn. 13, animals normally count as animate nouns in Niya Prakrit.


The sentence in which the exceptional animate loc.sg *maṃnuśami* ‘man’ occurs is given in (17). The basic message here is that Katg̲eya has become entitled to treat the man he bought (*maṃnuśami*) in whatever way he wants (*sarva karaṃnena*), i.e. essentially as movable property.34See Agrawala (1953) for some historiographical notes on this.

(17)

**Table:** 

*aja*	*kṣ̄una*	*uvatae*	***eda***	***maṃnuśami***
now	time.dir.sg	starting.from	**this.** **acc.sg**	**man.** **loc.sg**
*Katg[e]yas̱a* 35	*eśvarya*	*siyati*	*sarva*	*karaṃnena*
Katg̱eya.gen.sg	lordship.dir.sg	be.opt.3sg	all.dir.sg	doing.ins.sg
‘From now on Katg̲eya shall have ownership **over this man** for all matters.’				
(CKD 324 + 328 uo8–cr1; tr. after Burrow 1940: 61–62)				

35 Reading proposed in Schoubben (in prep.); previously read as *Katg̲ayas̱a*.

I therefore propose that the loc.sg *maṃnuśami* ‘man’ can be seen as an instance whereby a noun is conceptualised differently from its preferred animacy value because of the context in which it occurs.36Compare de Swart, Lamers, and Lestrade (2008: 136): “despite strong preferences for a certain animacy value of nouns, speakers may conceptualize nouns differently from this preferred value in different contexts” (see also Lahiri 2021: 188). Note further Santazilia (2023: 33): “Additionally, the speaker can **promote** or **demote** humans and other entities, that is to say, they can show more or less empathy, according to their beliefs or broader cultural factors, or by using certain discursive or temporary resources” (emphasis in the original). Thus, I would argue that the word for ‘man’ was put here in the synthetic locative case in order to encode linguistically this man’s lack of agency.37
Jamison (2000: 75) similarly observed that words for females and female PNs can be “assimilated to livestock in the animacy hierarchy” in sentences referring to marriage practices, where the female is “less capable of functioning agentively than her spouse”. On a different note, it seems that scribes were sometimes also confused as to whether they should use a synthetic locative or the postposition *vaṃti* as the complement of *eśvarya* ‘lordship’ + copula, since there are also three instances where *eśvarya* + copula selects *vaṃti* despite the complement being the prototypically inanimate noun *bhuma* ‘land’ (see fn. 25).


Similar to what I did in Section 3.2 for the postposition *paride* ‘from’ and the synthetic ablative, I shall end this subsection with pairs of example sentences where the same verb selects a synthetic locative for an inanimate complement and the postposition *vaṃti* ‘in, at, to’ for an animate one. Examples (18a) and (18b) concern the phrasal verb *asaṃna gach*- ‘to take possession of’ (Skt. *āsannam* + √*gam*; cf. Konow 1938: 155).38For further examples of *asaṃna gach*- ‘to take possession of’ + *vaṃti* + animate complement, cf. CKD 256 cr1; 325 r2; 436 cr1–2; 494 cr2–3; 503 r1; 573 cr3; 588 uo5–6; 621 o7; 629 r1–2; 770 uo7–8; 788 co1–3; 817 B4; 881 cr5–6; 884 uo11; 898 6–7. In CKD 235 cr1, *asaṃna gach*- is used with abl.sg *tade* ‘from that’.

(18a)

**Table:** 

*adharmena*	*draṃgadharanaṃ*	***taya***	***striae***
against.the.law	law.official.gen.pl	**this.** **gen.f.sg**	**woman.** **gen.sg**
***vaṃti***	*na*	***asaṃna gaṃdavo***	
**in, at, to**	neg	**take.possession.of.** **ger**	
‘Against the law officials **must** not **take possession of that woman**.’			
(CKD 3 uo4; tr. Burrow 1940: 1)

(18b)

**Table:** 

***bhumaṃmi***	*Caklas̱a*	***asaṃna gaṃdhavo***
**land.** **loc.sg**	Cakla.gen.sg	**take.possession.of.** **ger**
‘… Cakla **must take possession of the land**.’		
(CKD 624 o4; tr. Burrow 1940: 130)		

In (19a) and (19b), we have examples with the light verb construction *cita kar*- ‘to pay attention’ (Skt. *cintām* + √*kr̥*).39More examples of *cita kar*- ‘to pay attention’ with an explicit, non-pronominal complement are found in CKD 349 o5 (with *vaṃti*) and CKD 165 o10; 320 r1 (with the synthetic loc).

(19a)

**Table:** 

*avi*	*edas̱a*	*Sag̱amoyas̱a*	*maṃtra dadavo*
also	this.gen.sg	Sag̱amoya.gen.sg	tell.ger
***uṭiyāna***	***vaṃti***	***cita kariṣyati***	
**she-camel.** **gen.pl**	**in, at, to**	**pay.attention.fut.3sg**	
‘This Sag̱amoya should moreover be told (that) **he should pay attention to the she-camels**.’			
(CKD 840 cr2–3; tr. mine)

(19b)

**Table:** 

*tahi*	*Lýimsuas̱a*	***eda***	***karyami***
you.gen./dat.sg	Lýimsu.gen.sg	**this.** **acc.sg**	**business.** **loc.sg**
***cita kartavo***			
**pay.attention.** **ger**			
‘By you, Lýimsu, **attention is to be paid to this matter**.’			
(CKD 140 co3–5; tr. Burrow 1940: 25)			

In (20a) and (20b), the verb used is *nikhas*- (Skt. *niṣ* + √*kas*), which literally means ‘to go out’ but which can also be used in the technical sense ‘to be spent/disbursed’.40For additional instances of *nikhas*- combined with a locatival complement, see CKD 478 o1–2; 612 r1; 685 *passim* (with *vaṃti*) and CKD 637 o3–5; 788 uo10 (with the synthetic loc). For the semantic change of ‘to go out’ to ‘to be spent/disbursed’, see Schoubben (fthc.: Appendix) and the references cited there.

(20a)

**Table:** 

***cojhbo***	***Naṃtipalas̱a***	***vaṃti***	*aṃna*	*nisag̱a*
***cojhbo*.** **dir.sg**	**Naṃtipala.** **gen.sg**	**in, at, to**	corn.dir.sg	maintenance.dir.sg
***nikhasta***				
**go.out.pst.3sg**				
‘Corn for maintenance **went out [i.e. was disbursed] to the *cojhbo* [official title] Naṃtipala [PN]**.’				
(CKD 478 o3; tr. mine)

(20b)

**Table:** 

*uṭana*	***yaṃñeṣu***	***nikhastaṃti***
camel.gen.pl	**sacrifice** **.loc.pl**	**go.out.pst.3pl**
‘The camels **went out to the sacrifices**.’		
(CKD 637 o11; tr. Burrow 1940: 133)		

In brief, for both of its spatial cases, Niya Prakrit makes a consistent distinction between synthetic and analytic case marking based on animacy. The preceding discussion of this distinction has focused mainly on nouns; but the animacy criterion is also relevant for the morphosyntax of personal pronouns, as will be argued in Section 5.

## 5 Animacy and the paradigm of the personal pronouns

As discussed in a recent article (Schoubben 2022: 3–7), the paradigm of the first and second person pronouns in Niya Prakrit consists of a threefold system with a direct case form (e.g. dir.sg *ahu* ‘I’ < *aham*); a genitive/dative case form (e.g. gen./dat.sg *mahi* ‘I’ < *mahya(m)* and gen./dat.sg *mama* ‘I’ < *mama*); and some remnants of instrumental case forms (e.g. ins.sg *maya* ‘I’ < *mayā*). What has not been discussed in that article is the complete lack of ablatives or locatives in the paradigm of the first and second person pronouns. This absence is noteworthy, though, given that other MIA languages, including Apabhraṃśa, do inflect personal pronouns in the ablative and locative.41For the personal pronouns in Pāli, see Oberlies (2019 [2001]: §§49–52, pp. 264–278); for most Prakrits, von Hinüber (2001 [1986]: §§365–373, pp. 251–254); and for Apabhraṃśa, De Clercq (2009: 61–65).


We can now account for the lack of such forms in Niya Prakrit as another instance of animacy governing the case system. Since first and second person pronouns are animate by necessity (cf. e.g. Santazilia 2023: 22–23), they are expected to have lost synthetic forms for the ablative and locative in a language that restricts those to inanimate nouns. Indeed, the postpositional constructions with respectively *paride* ‘from’ and *vaṃti* ‘in, at, to’ are attested with pronominal complements in the genitive,42With *paride* are attested: *asmakaṃ paride* ‘from us’ (CKD 690 r6); *tahi paride* ‘from you (sg)’ (CKD 139 o3; 201 o4; 307 o3; 368 o5; 390 o4; 399 rA2; 541 o4 = r4); *tava paride* ‘from you (sg)’ (CKD 161 uo6); *tumahu* (*tumaho*) *paride* ‘from you (pl)’ (CKD 164 o3; 494 uo2; 690 r7). With *vaṃti* we find: *mahi vaṃti* ‘in/at/to me’ (CKD 152 cr4; 317 o3; 612 r1); *tahi* (*tehi*) *vaṃti* ‘in/at/to you (sg)’ (CKD 288 uo3; 373 r3; 594 o3); *tumaho vaṃti* ‘in/at/to you (pl)’ (CKD 320 r1–2, r6). thus supplying an analytically formed ablative and locative for the personal pronouns. Such a distinction between core cases and peripheral cases in the paradigm of personal pronouns is also typologically common. The Australian language Dyirbal, for instance, is another language where pronouns cannot be put in the ablative or locative case because the latter are restricted to inanimates (Aristar 1997: 319, 337; Dixon 2022: 30).

Third person pronouns can be either animate or inanimate, and for these, synthetically formed ablatives and locatives do occasionally occur in Niya Prakrit. Since, however, locatives are rare, and not much can be added to Burrow’s discussion of them (1937: §§80–82, pp. 33–35),43I have been unable to find a Niya example of the loc.pl *teṣu* ‘among those’ cited by Burrow (1937: §80, p. 34). I shall focus here on ablatives.

To begin with, some comments are due on *tade*, the synthetic abl.sg of the 3rd person pronoun *sa*-/*ta*- ‘this’ (Burrow 1937: §80, pp. 33–34). Leaving badly intelligible examples44CKD 394 o2; 414 r2; 632 o4; 806 A6, A7. The examples in Kuča Prakrit (CKD 869 4, 5, 8; 883 r2) have been put aside as well (cf. fn. 4). and cases where *tade* can just mean ‘then’ (cf. Skt. *tataḥ*) aside,45CKD 4 uo2; 367 o2; 403 uo2; 436 uo4; 482 uo2; 519 o6; 573 uo7.
*tade* normally means ‘from it’ or ‘from there’.46For a list of the relevant attestations, see Appendix 2a. I have found two exceptional cases (CKD 272 o7; 479 cr1) where *tade* means ‘from him’ (for which *tas̲a paride* or the like is normally used; see just below). Like nouns inflected in the ablative case (Section 3.2), *tade* is thus inanimate by definition; and when 3rd person pronouns have animate referents, the postposition *paride* ‘from’ is used to express ablatival relations, e.g. *tasa* (*tas̱a*) *paride* ‘from him’ (CKD 140 cr5; 719 o4) or *edeṣa paride* ‘from them’ (CKD 189 o2). In one special construction, i.e. when *tade* is used in a partitive sense dependent on the numeral *eka* ‘one’ or the adjective *avaśiṭha-* ‘the remaining’, *tade* seems indifferent to both gender and number; compare *tade … eka* ‘one of those’ (CKD 106 uo7), *tade eka arivag̱a* ‘one guide from those’ (CKD 253 r1), *tade avaśiṭhe* ‘the rest of those (children)’ (CKD 279 o3), *tade eka uṭa* ‘one camel of these’ (CKD 359 r1), and *tade vaḍ̱avi 1* ‘one mare from them’ (CKD 509 uo3). *paride*, by contrast, is not found governed by a numeral, which may explain why *tade* is used for animates (and plurals) in this particular construction.

The usage of *imade* (also *iṃmade*), the synthetic abl.sg of the near-deictic pronoun *i*-/*ima*- ‘this’ (Burrow 1937: §82, pp. 34–35), is more straightforward: it is always used in an adverbial sense ‘from here’ and thus inanimate by definition.

Much the same is true about the adverbially used *adehi* (also *atehi*) ‘from there’ (Burrow 1937: §91, pp. 40–41), which supplies a far-deictic equivalent to *imade*. Etymologically, *adehi* consists of *ade < ataḥ*, abl.sg to the pronoun *a*- ‘that’, plus an element -*hi*, thus parallelling (Jaina-)Māhārāṣṭrī *iohi* ‘from here’ < *itaḥ* + -*hi*.47The Māhārāṣṭrī form is cited by Ollett (2022: 275) from the *Taraṅgalolā*. The etymological origin of the element -*hi* is debated. Ollett compares the Prakrit abl.sg in -*āhi* of the type *mūlāhi* ‘from the root’ which he derives from abl.sg *mūlāt* + the particle *hi* ‘for’. For these Prakrit ablatives, I prefer, however, the explanation of Insler (1991–1992), who interprets them as relics of the Vedic usage of ablative + semantically empty *ádhi* ‘above’, thus *mū́lāt ádhi > *mūlā ahi > *mūlāhi* (for the Vedic usage, see also Hettrich 1991: 56–58). As for Niya *adehi* and Mahārāṣṭrī *iohi*, I would suggest to explain these in the same vein as Insler did for the Prakrit abl.sg in -*āhi*: *adehi* can be compared with the Vedic syntagm *átó ’dhi* ‘from there; from that point onwards’ < *átaḥ* + *ádhi* (as in e.g. AVŚ 6.111.1c = AVP 5.17.6c or MS 1.4.5; cf. Amano 2009: 143 fn. 21); and *iohi* with Vedic *ito ’dhi* ‘from here’ < *ítaḥ* + *ádhi* (as in e.g. ChUp 5.3.2).


## 6 A note on Khotan Prakrit *sag̲aj̲i* ‘in, at, to’

As already noted in Section 1 (fn. 4), Niya Prakrit is not the only known dialect of Central Asian Gāndhārī; in addition, we have Kuča and Khotan Prakrit. In the former, the postpositions discussed in this article are attested as well, but neither has so far popped up in Khotan Prakrit.48In Kuča Prakrit, there is one attestation of *paride* ‘from’ (spelled *parite*; CKD 838 o2) and three of *vaṃti* ‘in, at, to’ (CKD 832 4; 837 A5; 838 r3). The postpositions are completely absent from South Asian Gāndhārī (for *vaṃti*, this had previously been noted by Salomon 1998: 79). Because of the fairly limited corpus available for this dialect (CKD 661; 843), this may be due to chance. With Noble (1931: 453), I would, however, like to observe that in (21) the Khotan Prakrit postposition *sag̲aj̲i* /saγāźi/ occupies the same syntactic slot as *vaṃti* does in Niya (and Kuča) Prakrit. The noun phrase governed by *sag̱aj̱i* encodes the recipient (∼ a dative) of the prs.1sg *vikrināmi* ‘I sell’, just like in (13) a noun phrase governed by *vaṃti* was used to express the recipient of pst.3pl *vikridati* ‘they sold’.

(21)

**Table:** 

*ta*	*idani*	*so*	*uṭo*	***vikrināmi***	*mulyȧna* 49	
thus	now	this.acc.sg	camel.acc.sg	**sell.prs.1sg**	price.ins.sg	
*maṣȧ*						
bronze.coin.dir.pl						
*sahasra*	*aṣṭi*	*4 4 1000*	***Sulig̱a***	***Vag̱iti***	***Vadhag̱asya***	
thousand	eight	8000	**Sogdian.** **dir.sg**	**Vag̱iti.** **dir.sg**	**Vadhag̱a.** **gen.sg**	
***sag̱aj̱i***						
**in, at, to**						
‘Thus now **I am selling** this camel for a price of 8000 bronze coins **to the Sogdian Vag̱iti Vadhag̱a**.’						
(CKD 661 o3–4; tr. after Burrow 1940: 137)						

49 Note that ⟨ȧ⟩ indicates a palatal vowel in Khotan Prakrit.

Another parallel between Khotan Pkt. *sag̱aj̱i* and Niya Pkt. *vaṃti* lies in their etymology. As discussed in Section 4.1, *vaṃti* derives from the OIA relational noun *upānte* ‘in the proximity of’, loc.sg of *upānta*-; similarly, *sag̱aj̱i* goes back to the OIA relational noun *sakāśe*, loc.sg of *sakāśa*-, also meaning ‘proximity’. Compare further the Pāli postpositions *santike* ‘in the presence of > with, before’ and *samīpe* ‘in the proximity of > near’, cited in Section 4.1.

While dependent on the discovery of additional Khotan Prakrit materials, it thus seems possible that the expression of the locative case for animate nouns is an isogloss separating Niya from Khotan Prakrit (cf. also Schoubben 2024: 402–403; 523–526).

## 7 Concluding remarks

To summarise, the Niya Prakrit postposition *paride* ‘from’ has been grammaticalised as a marker of ablatival relationships and is always accompanied by a genitive complement right in front of it. This complement being animate by default, *paride* fulfils the role of an animate ablative in the language, whereas the synthetic abl.sg in -*ade* (-*āde*)50As noted in Section 2, no abl.pl is attested so far. has come to be restricted to inanimate nouns. In the same way, the postposition *vaṃti* ‘in, at, to’ should be characterised as a marker of animate locatives, while the synthetic loc.sg in -*aṃmi* and the loc.pl in -*eṣu* typically select inanimate complements. As a result, the paradigm of the Niya Prakrit *a*-declension given in Section 2 only applies to inanimate nouns such as *bhuma*- ‘land’ and not normally to animate nouns such as *maṃnuśa*- ‘man’ (< OIA *manuṣya*-), whose ideal paradigm would rather be as follows:

**Table:** 

	sg	pl
dir	*maṃnuś-a*	*maṃnuś-a* (-*e*)
ins	*maṃnuś-ena*	*maṃnuś-ehi*
gen	*maṃnuś-as̲a* (-*asya*)	*maṃnuś-ana* (-*anaṃ*)
abl	*maṃnuś-as̲a* (-*asya*) *paride*	*maṃnuś-ana* (-*anaṃ*) *paride*
loc	*maṃnuś-as̲a* (-*asya*) *vaṃti*	*maṃnuś-ana* (-*anaṃ*) *vaṃti*

Consequently, one can say that the direct, the genitive, and the instrumental are the three core cases in Niya Prakrit, while the ablative and the locative seem to be peripheral.

The pervasive role animacy plays in the nominal morphology of Niya Prakrit makes this language an outlier in the spectrum of MIA languages we know of. As briefly hinted at in Section 3.1, Reinöhl found that the outcomes of *madhye* ‘in the middle of’ and *upari* ‘above, on top’ only inconsistently select animate complements in Pāli and Apabhraṃśa, and that the grammaticalisation process towards case markers was fully completed only in 16th-century Old Avadhī. Thus, we seem to have here another instance where Niya Prakrit parallels developments found in other Indo-Aryan languages, “but at a much earlier period” (Jamison 2000: 65).

Sometimes, grammatical differences between Niya Prakrit and other MIA languages can be attributed to contact with neighbouring languages (cf. Schoubben 2024: Ch. 5). Yet, in the present case, no convincing source seems to be at hand. Animacy plays a certain role in the case system of Tocharian A and B, where there is differential object marking (DOM) in that the obl.sg of thematic nouns only ends in -*ṃ* when the referent is [+human] (e.g. TA *oṅkaṃ* ‘man’; TB *eṅkweṃ* ‘id.’).51
Akao (2020: 12–13) compares the Tocharian DOM to the Old Turkic plural suffix -*lAr*, which is by default used for humans and mythological figures, and considers this to be a further argument for language contact between Tocharian and Turkic. The parallel identified by Akao is, however, far from exact, and I rather agree with Peyrot (2019: 94–95) that the Tocharian DOM results from language-internal developments. This, however, is hardly to be compared to the Niya Prakrit ablative and locative.52Note also that structural influence from Tocharian on Niya Prakrit remains to be convincingly demonstrated in any case (Schoubben 2024: Ch. 6). In Bactrian, too, an exact *comparandum* is lacking; in Kuṣāṇa Bactrian, plural nouns governed by prepositions take the obl.pl ending in -ανο /ānə/ for animates and the dir.pl ending in -ε /e/ for inanimates (Sims-Williams 2004 [2008]: 66), but this is again too different from the Niya Prakrit pattern to have been its source.53At a more general level, it has been proposed that contact with Dravidian languages may have contributed to the extension of postpositions in Indo-Aryan. Dravidian languages are, however, not the most obvious source for a grammatical feature in Niya Prakrit, and the whole idea of Dravidian influence on the Indo-Aryan case system needs further study anyhow (cf. Reinöhl 2016a: 120–122, 2016b: 123 fn. 38). There are some phraseological parallels between Niya Pkt. *paride* ‘from’ and Bactr. ασο ‘id.’: compare, e.g., Niya Pkt. *yo iśa vartamāna Lýimsuas̱a paride ñadartha bhavidavo* “What news there is here you will learn from Lýimsu [PN]” (CKD 161 o12–13; tr. Burrow 1940: 32) with Bactr. οδο σιδμο μισο – πιδοχοανο τα[δο το ϸιγ]ανο ασο ϸαοροχωζο πηδανο βο[ο] “And whatever my further news, [your excelle]ncy will be informed by Shahr-khoz [PN]” (**kc**17–19; Sims-Williams 2025: 18); but, in contrast to *paride*, Bactr. ασο does occur with inanimate complements.


If not language contact, which other factor may account for the Niya Prakrit case system behaving differently from that of other MIA languages? I submit that it is the difference in text genre. Niya Prakrit is known from administrative documents, whose language is “remarkably varied and flexible” (Jamison 2000: 64).54An anonymous reviewer of this paper wondered whether analytic case marking could also be felt to be more explicit or precise, and therefore more suited for legal and other bureaucratic texts. This hypothesis seems possible to me, but it would not explain why the analytic case marking is specifically used for animate nouns. Other MIA languages are mainly attested via literary compositions – texts whose language does not normally reflect the spoken vernacular (cf. Masica 1991: 51), and where influence from prestige languages like Sanskrit and Māhārāṣṭrī often obscures linguistic developments (cf. Bubeník 1998: 16–19).55Of course, I do not want to imply that administrative documents reflect the spoken language perfectly or that Niya Prakrit was immune to influence from Sanskrit, but there is a clear difference between administrative and literary texts. If, then, postpositions are not yet fully integrated into the case system of these MIA languages, including Apabhraṃśa, and if animacy plays a less decisive role than in Niya Prakrit and in NIA, this may tell us more about what authors wanted the language of their literary texts to look like than about the actually spoken vernacular.56
Reinöhl (2016a: 136) briefly refers to end rhyme as a factor for word order patterns in Apabhraṃśa, but otherwise, she largely ignores the *Kunstsprache*-like character of this language (for which see e.g. Tieken 2000: 59–61). In other words, postpositional case marking could have come into being at a (much?) earlier point in the history of Indo-Aryan than the evidence from literary Prakrits would suggest.

## Text-critical marks in the Prakrit quotations 57 These marks have been taken over from Baums and Glass (2002–).

[ ]: uncertain reading
(*): editorial restoration of lost text
⟨*⟩: editorial addition of omitted text
?: illegible akṣara
+: lost akṣara
///: textual loss at left or right edge of support

## Abbreviations 58 Grammatical abbreviations included in the Leipzig Glossing Rules have not been listed below.

AVP: Atharvaveda Paippalāda
AVŚ: Atharvaveda Śaunakīya
Bactr.: Bactrian
ChUp: Chāndogya Upaniṣad
CKD: Corpus of Kharoṣṭhī Documents
co: cover-tablet obverse
cr: cover-tablet reverse
dir: direct case
DOM: differential object marking
ger: gerundive
MIA: Middle Indo-Aryan
MS: Maitrāyaṇī Saṃhitā
NIA: New Indo-Aryan
o: obverse
OIA: Old Indo-Aryan
opt: optative mood
Pkt.: Prakrit
PN: personal name
r: reverse
Rām.: (Vālmīki) Rāmāyaṇa
Sgh.: Saṅghāṭasūtra
Skt.: Sanskrit
TA: Tocharian A
TB: Tocharian B
uo: under-tablet obverse
ur: under-tablet reverse
v.l.: *varia lectio*

## Appendix

### 1. Attestations of the Niya Prakrit postposition *paride* ‘from’ 59 I leave aside CKD 77 r4; 113 r3; 644 r1; 885 co1 because they are fragmentarily preserved and/or contextually unclear and thus not usable for the linguistic investigation undertaken here.

CKD 7 uo2; 11 uo2; 16 uo2; ; 60 Here and in what follows, attestations that formed the basis for the example sentences above have been put in a frame. 39 cr1; 54 o2; 86 r5; 88 r1; 97 o5; 100 o3; 125 o1; 126 o4; , o5; 140 cr5; 157 r1; 159 r1; ,  cr6; 164 o3; 165 o8, o13; 189 o2; 195 + 726 co1, o4; 201 o4; 208 o1; 222 + 336 co1; 252 o1; 272 o3; 288 cr3, cr4; 290 rD3; 291 o5; 297 cr2 (2×); 307 o3; 312 uo2; 318 uo5; 325 o1; 330 o2; 331 co1; 335 co1; 339 o2; 345 co1, ; 359 r1; 364 r1; 368 o5; 370 cr1; ; 385 o4 (4×), o5; 390 o4; 399 rA2; ; 418 o3; 419 co1; 421 o1; 422 o5; 425 co2; 431 uo5 = 432 uo3; 431 cr2 = 432 uo7; 433 uo2; ; 468 uo2; 494 uo2; 498 o5; 500 o2; 506 o3; 507 o2; 508 o5; 516 o3; 524 uo2; 536 oA1, oA2, oA3, oA4, oB1, oB2, oB3, oB4, oB5; 539 o1; 541 o4 = r4, o6 = r6; 546 o2, o3, o4, o8; 553 o2; 561 uo3; 562 cr1; 568 co1; 569 co1, uo3; 570 co1, cr1; 571 uo4; 574 uo2, uo3, , cr2; 575 co1, uo7 (2×), uo8; 576 co1; 577 co1, co3; 578 ur1; 580 uo3; 582 uo3; 583 uo4; 584 uo6; 588 co1; 590 uo2; 591 uo2; 593 co2, uo3; 630 o2, o4; 633 r7; 675 o2; 677 uo2; 678 uo3; 690 r6 (2×), r7; 717 o1; 719 o4; 735 r1; 749 o1; 788 cr1; 792 co1; 793 A1; 795 co2, uo2, uo4; 797 cr3; 61 Instead of *Votug̱eyas̱a paride* ‘from V.’, read in this line *Sotug̱eyas̱a paride* ‘from S.’ (cf. *Sotug̱eyas̱a vaṃti* ‘to S.’ in CKD 122 o2), with Hasuike (1996: 308). 813 1; 820 uo3; 841 uo2; 884 co1; 889 co1 **∼ 149 instances**.

### 2. Attestations of the synthetic ablative

#### a. Attestations of *tade* ‘from that; from there’ (cf. also fn. 44, 45)

CKD 17 uo3; 24 uo4; 31 + 764 cr1; 62 co1; 71 cr2; 140 cr1 (2×); 156 uo3; 165 o3, o5; 198 o2 (‘from there’, *pace* Burrow’s 1940: 37 ‘from him’); 212 uo3; 225 o7; 235 cr1; 236 uo2; 244 r2; 272 o3, o7 (‘from him’); 278 r2, r5; 283 o3; 295 o3; 305 o5, o7; 309 o2; 312 cr1; 341 o3; 357 o6; 373 r1, r2; 375 o3; 383 r6; 387 o3; 399 o4 (2×), o5; 400 o2; 401 uo2 (‘from there’; left untranslated in Burrow 1940: 82); 430 uo2; 434 uo3; 437 uo4; 479 cr1 (‘from him’); 480 cr1; 482 uo4; 499 o6; 502 o2, o4; 517 o2; 530 uo3; 562 cr2; 570 uo6; 574 uo5, cr1; 582 uo3, cr7; 584 uo6; 621 o3; 706 uo2; 709 uo6 (spelled *tate*), uo7, cr2; 735 r3 (2×); 760 o2; 796 cr2; 801 uo2; 819 uo3, cr1; 881 uo4; 884 uo6, uo7.

#### b. Nominal attestations of the abl.sg in -*ade* (-*āde*) 62 The following attestations have been left out of consideration because their reading and/or semantics are too uncertain: *apñighade* (CKD 417 o2); *asaṃ kicade* (CKD 869 8); *ukastade* (CKD 40 uo3); *thavatade* (CKD 869 3); *panicanade* / *paniṃcanade* (CKD 100 o1; 140 cr3); *storade* (CKD 805 cr7).

***aṃnade*** ‘corn’: CKD 140 cr1; ***acoade*** ‘border’: CKD 152 cr1; ***anatiyade*** ‘command’: CKD 562 uo3; ***Apg̱eyade***, PN: ; ***ardhade*** ‘half’: CKD 315 uo2; ***av̱anade*** ‘village’: CKD 334 r1; 573 uo4; 696 o5; 817 B3, C1; ***asiyade*** (*as̱iyade*) ‘mouth’: CKD 63 uo4; 90 o2, o3; 144 uo3; 184 r1; 255 o2; ***utarade*** ‘north’: CKD 839 uo9; ***udag̱ade*** ‘water’: CKD 502 o2, o4; ***upajivade*** ‘dependence’: CKD 770 uo5; ***ubhayaṃdade*** ‘both sides’: CKD 387 o2; ***kilmeyade*** (*kilmiyade*) ‘family estate’: CKD 358 o6, o7; 817 B4; 884 uo4; ***kriṣide*** ‘ploughed field’: CKD 140 uo6, cr2; ***Krorayinade***, toponym: CKD 696 o3; ***Khaṃniyade***, toponym: CKD 722 cr7; ***khalade*** ‘threshing floor’: CKD 140 cr4; ***Khotaṃnade***, toponym: CKD 248 o2; 272 o2, o4, o9; 283 o2; 289 o2; 291 o2; 329 o2; 333 o2; 341 o2; 349 o3; 351 o3; 357 o2; 358 o2; 362 o3, o6; 379 o2; 400 o2; 403 uo2; 578 ur3; 584 cr2; 637 o7; ***khvaniyade*** (*khvaniyāde*; *khvaniyaṃde*; *kuhaniyade*) ‘capital’: CKD 478 o1; 506 o6; 660 oA1; 663 o2, o3, o4; ***gadag̱ālade*** ‘time of arrival’: CKD 660 oA1; ***garbhaśalyade*** ‘pains of childbirth’: CKD 702 o5; ***goṭhade*** ‘farm, household’: CKD 36 uo2; 71 uo3; 107 o4; 165 o6; 211 r3; 216 r4; 224 r1; 295 o4; 331 uo5; 345 uo4; 366 o2; 387 o9; 621 o3; 625 o4; 643 o2; 706 uo2, uo4; 713 o8, o9; 714 o7, o8; 823 r4; 842 o8; 881 cr4; ***Caḍ̱odade*** (*Caḍ̱otade*; *Coḍ̱otade*), toponym: CKD 14 cr1; 214 o3, o4; 236 uo2; 246 r5; 305 o4; 306 r1; 367 o2–3; 532 uo4; 575 uo2; 842 o3, o4, o9; 63 In CKD 842, read *Caḍ̱otade* / *Coḍ̱otade* instead of Zhang’s (2013: 214–215) *Caḍotade* / *Coḍotade*.
; ***Calmadanade***, toponym: ; 362 o4; 722 uo6; ***Cinasthanade*** ‘China’: CKD 35 r1, r2; ***taḍ̱itag̱ade*** ‘beating’: CKD 144 co3; ***tanuvag̱ade*** (*tanuv̱ade*) ‘one’s own (property)’: CKD 305 o5, o7; 635 r5; ***dakṣ̄iṃnade*** ‘south’: CKD 839 uo8; ***draṃgade*** (*traṃghade* 64 For this reading, see Schoubben (2024: 188 fn. 426). ) ‘office’: CKD 317 o6; 640 o1; ***dharmade*** ‘office, duty’: CKD 381 o2; 435 uo3; 567 uo4; ***nagarade*** ‘city’: CKD 55 uo2; 69 o4, o5; ***nagaraprichade*** ‘investigation in the city’: CKD 504 o2; ***Ninade*** (*Nināde*), toponym: CKD 14 cr1, cr2; 637 o7; ***paṃthade*** ‘road’: CKD 310 uo5; ***paḱayade*** ‘share’: CKD 608 o1; ***padade*** ‘foot’: CKD 358 o6; ***padamulade*** (*pādamulāde*) **‘**root of the feet’: CKD 24 uo3; 637 o7, o9, o11; 696 o6; 702 o3; 885 uo2; ***parabulade*** ‘fort’: CKD 415 uo6; ***paraviṣeyade*** ‘foreign territory’: CKD 884 uo9; ***parikrayade*** ‘hire’: CKD 52 uo3; ***parvatade*** ‘mountain’: CKD 634 o6; 635 r1; 637 o5; ***pac̄imade*** ‘western’: CKD 839 uo9; ***Pis̱aliyade***, toponym: CKD 64 o3; 341 o4 (2×); ***Puṃniyade***, toponym: CKD 554 o1; ***purastimade*** ‘east’: CKD 839 uo8; 65 My reading; Duan (2013: 226) reads this word as *purāthimade*.
***purvadiśade*** ‘eastern direction’: CKD 90 o4; ***Puṣg̱ariyāde***, toponym: ; ***potg̱eyade***, a type of water reservoir: CKD 120 o3; ***prathade*** ‘journey’: CKD 152 uo7; ***pradej̱ade*** (*pradej̱ate*) ‘region’: CKD 41 o3; 79 r1; 173 oB1; 175 rB1; 277 oA1, r1; 304 oA1; 631 oA1; ***bahiyade*** ‘outside’: CKD 222 + 336 uo3; 345 uo8; ***bhagade*** ‘part’: CKD 211 r5; ***bhumade*** ‘land’: CKD 331 uo3; 574 uo5, ; ***miṣiyade*** ‘agricultural land’: CKD 296 uo2; 808 uo6; ***mulade*** ‘root’: CKD 160 o6; 271 + 338 cr4; ***rajade*** (*rayade*; *rajyade*) ‘state, province’: CKD 22 o3; 164 o10; 182 o2; 223 uo2, uo3 (2×); 272 o7; 351 o5; ; 392 o4; 402 o1; 667 r5; 714 o5, o5; 760 o1; ***rayakade*** ‘royal (office)’: CKD 399 o4; 600 o2; 640 o1; ***rayakulade*** ‘royal herd’: CKD 840 cr4; ***rayadvarade*** (*rajadvarade*; *rayatvarade*) ‘royal palace’: CKD 8 o1; 159 oB1; 246 r4; 392 o3; 540 uo3; 578 ur5; 788 uo5; 881 uo2; ***rocakhorade***, a type of location: CKD 320 r7; ***lekhade*** ‘letter’: CKD 376 o7; 637 o12; ***vacanade*** ‘command’: CKD 396 o4; ***varṣade*** ‘year’: CKD 226 o3; ***viṃśpade***, administrative unit: CKD 82 o1; ***viloṭade*** ‘looting’: CKD 494 uo3, cr1, cr2; ***vivatade*** ‘dispute’: CKD 540 uo3; ***śaḍ̱ade*** ‘vineyard’: CKD 574 co1, cr4; ***śatade*** (*śadade*) ‘hundred [an administrative unit]’: CKD 82 o1; 94 r24, r29, r35, r43; 115 oA1, oC1–D1, oE1, oG1, oI1–K1, oL1; 132 rA1, rB1, rC1, rD1, rE1, rF1, rG1, oA1, oB1, oC1, oD1, oE1, oF1, oG1; 342 oA2, oA3, oA4; 650 rA1, rB1, rC1, rD1, rE1; 701 rA1, rB2, rC4, rD1, rD7, rF1, rG4, oA1, oC1, oE1; ***śavathade*** (*śavatade*) ‘oath’: CKD 51 r1; 527 o4; 576 uo4; ***Sacade*** (*Sācade; Sachade*)*,* toponym: CKD 14 uo3; 123 o1; 133 o3; 159 oB2; 214 o3 (2×); 306 r1; 367 o2; ***Samarsade***, toponym: CKD 64 o2, o3; ***simade*** (*siṃmade*) ‘border’: CKD 147 oA17, oB1; 531 oB5; 544 oC3; ***Supiyade***, toponym (cf. fn. 15): CKD 515 uo3; 722 uo6; ***Suryadade***, toponym: CKD 572 cr1; ***senade*** (*seniyade*) ‘army’ (cf. fn. 15): CKD 399 o5; 562 uo2; ***Seniyade***, toponym: CKD 580 cr1; ***S̄unade***, toponym: CKD 64 o3 (2×); ***hastade*** ‘hand’: CKD 163 r3; 216 r1. **∼ 77 types; 273 tokens.**

### 3. Attestations of the Niya Prakrit postposition *vaṃti* ‘in, at, to’ 66 I leave aside CKD 100 r6; 672 oB1; 722 co4; 771 uo7; 882 B3 because they are fragmentarily preserved and/or contextually unclear and thus not usable for the linguistic investigation undertaken here. Jiang’s (2020: 135) reading *vaṃti* in CKD 897 o1 is mistaken and has therefore been left aside as well.

CKD ; 5 uo2, uo3, cr1 (2×); 15 uo2; 19 uo2, uo3; 23 o2; 24 uo2; 39 uo2; 70 uo2 (cf. Burrow 1940: 15), uo3; 89 o6; 106 cr1, cr2; 118 rC1; 122 o2, o3; 127 r1; 130 uo4; 140 cr5 (2×); 152 uo5, cr4; 162 uo4; 170 oB1; 180 oA1, oA2, oA3, oA4, oA5, oB2, oB3, oB4, oB5, oB6, oB7, oB8; 186 o2; 187 o8 (2×); 209 o2; 212 uo3, cr2; 213 r1; 216 r2, r5; 226 o3; 244 r3; 247 o3; 256 cr1; 283 o5; ; 317 o3; 320 r2, r6; 322 uo8; 324 + 328 uo6, uo7; 325 r2; 327 o3; 345 uo12, cr1; 349 o5; ; 358 o7, o8; 359 r4; 373 r3; 401 uo2; ; 425 uo5, cr7; 436 uo5, cr2; 437 uo3; 478 o1, o2, ; 480 uo3; 492 uo2; 494 uo3, cr2, cr3; 502 o5; 503 r1; 505 o3; 524 uo4; 542 uo4; 546 o6, o7; 549 o2; 551 r1, r2; 555 r1; 564 uo2; 566 o3; 570 uo6; 571 uo2; 573 cr3; 575 uo3, uo4, cr3; 577 uo6; 578 uo4, uo6, uo7; 579 uo3, uo5; 580 uo2, uo7; 581 uo4; 582 uo2, uo4; 586 uo2; 587 uo2; 588 uo3, uo5; 589 uo2, uo4; 590 uo2, uo5; 592 uo2; 593 uo7; 594 o3; 612 r1; 621 o2, o7; 624 o2; 629 r2; 633 r5; 646 uo4; 648 o4; 654 uo2; 655 uo4; 656 uo6; 678 uo3; 683 o3, o4; 685 oA1, oA2, oA3, oA4, oA5, oA6, oB1; 686 oA2, oA3, oA4, oA6, oA7, oA8, oA9, oB1, oB2, oB4, oB5, oB6, o1; 703 r1; 709 cr3, cr4; 750 o3; 762 oD2, oD3; 770 uo8, uo10 (2×); 788 uo7, co1 (2×); 801 uo2; 817 B4, C1, C2; 820 uo8; 839 uo2; ; 841 uo3; 842 o7; 880 uo2, uo3; 881 cr5; 884 uo11; 889 cr2; 898 o7 **∼ 187 instances**.

### 4. Attestations of the synthetic locative

#### a. Nominal attestations of the loc.sg in -*a(ṃ)mi* (limited to CKD 1–400) 67 The following attestations have been left out of consideration because their reading and/or semantics are too uncertain: *+ + + + aṃḍaraṃmi* (CKD 16 uo3); *+ [a]riṣami* (CKD 25 o6); *tseg̱eyami* (CKD 30 o2); *uṣasaṃmi* (CKD 68 cr2); *[ko]liyaṃmi* (CKD 152 uo3); *aṃbaṃjayaṃmi* (CKD 252 r4); *yaṃbami* (CKD 265 uo2); *(protsa) kresaṃmi* (CKD 317 o7); *śa[dro]ḍ̱ami* (CKD 271 + 338 cr1).

***acovaṃmi*** ‘border’: CKD 125 o2; ***aṭhadaśami*** ‘eighteen’: CKD 354 o2; ***Ayamatuvasaṃmi*** *,* toponym: CKD 206 cr5, cr6; ***Alma Bhumiyaṃmi***, toponym: CKD 292 o4; ***av̱anaṃmi*** (*avanaṃmi*): CKD 10 uo2; 42 uo2, cr1, ur1; 46 uo2, uo3, ur1; 70 ur1; 105 oB1, oD1; 121 o2, o3; , ur1; 137 oA1; 163 o3; 165 o3; 181 r1; 193 r1; 199 oB1; 207 oa; 254 o2, r2; 275 o2, r1; 279 o2 (3×), o3 (2×), o4; 295 o2–3; 296 uo2, uo3, ur1; 297 uo2; 326 r2; 334 o2, r2 (2×), r3; 366 o2; 389 o2; 393 o2, r1; ***Astasaṃmi*** *,* toponym: CKD 124 uo2; ***kabhoḍhami*** (*kabhoḍhaṃmi*) ‘pasture’: CKD 13 uo2; 15 uo2 (2×), cr1; 392 o6; ***karyaṃmi*** (*karyami*) ‘work’: CKD 107 o4–5; ; 164 o6; 165 o10; 272 o2; 283 o2; 291 o2; 341 o2; 349 o2; 351 o2; 358 o2; 362 o2; 368 o2; ***kalaṃmi*** (*kālaṃmi*; *kāṃlaṃmi; kalami*) ‘time’: CKD 8 o1; 17 cr2; 33 uo2; 87 o1; 90 o1; 98 o1; 116 o1; 120 o1; 123 o1; 162 cr2; 209 o1; 215 o1; 225 r7; 228 o3–4; 305 o6–7; 322 uo6–7; 325 r1, r3; 331 uo7; 345 cr1; 348 uo1, uo4, cr1; 350 r2; 383 r5–6, r7, r10; ***kilamuṃtraṃmi*** ‘sealed wedge-tablet’: CKD 160 o4; ***kilmeyaṃmi*** ‘family estate’: CKD 32 uo2–3; 256 uo2; ***kuhaniyaṃmi*** ‘capital’: CKD 291 o3; ***Kog̱itsasaṃmi***, toponym: CKD 133 o3; ***kriṣiyaṃmi*** ‘ploughed land’: CKD 83 cr6; ***kriṣivatraṃmi*** (*kriṣivatrami*) ‘cultivation’: CKD 320 r1; 368 o3; ***Krorayinaṃmi***, toponym: CKD 15 uo2; ***kṣ̄unaṃmi*** (*kṣunami*) ‘date, reign, regnal year’: CKD 121 o1; 147 o1; 169 r1; 180 o1, oB1; 195 + 726 uo2; 203 r1; 204 o1; 209 o1; 222 + 336 uo2; 298 r1; 318 uo2; 321 o2; 322 uo2; 324 + 328 uo2; 327 o2; 331 uo2; 343 o1–2; 345 uo1; 350 o1; 369 o1–2; 378 o1; ***Khemaṃmi***, toponym: CKD 214 o5; ***Khotaṃnaṃmi*** (*Khotaṃnami*), toponym: CKD 14 uo2, cr1 (2×), cr2; 22 o2; 135 uo2, uo3; ; 223 uo2; 248 o6; 251 o2; 362 o3, o4–5; 367 o3; 388 o2; 400 o2; ***guj̲athanaṃmi*** ‘hidden place’: CKD 17 cr2; 68 Instead of *guṭathanaṃmi* ‘in a hidden place’, I read *guj̲athanaṃmi* ‘idem’, loc.sg of *guj̲athana*-* < *guhyasthāna*- (see Schoubben 2024: 21 fn. 42).
***goṭhaṃmi*** (*goṭhami*) ‘farm, household’: CKD 31 cr1; 152 cr2; 157 o5; 225 r2; 310 urA4; 331 uo5–6; 370 cr3; ***goniyaṃmi*** ‘bag’: CKD 214 o3, o4; ***gośaḍ̱aṃmi*** ‘cow enclosure’: CKD 157 o4; ***gramaṃmi*** ‘village’: CKD 310 urA3; ***Caḍ̱otaṃmi*** (*Caḍ̱otami*; *Caḍ̱odaṃmi*), toponym: CKD 27 uo2; 159 r3, r4; 183 r1; 271 + 338 uo2; 310 urB1; 340 o3; 69 In CKD 340 o3, I propose to restore *[ca] ? taṃmi uṭa ukasita* as *[Ca](*ḍ̱o)taṃmi uṭa ukasita* ‘the camel went away to Caḍ̱ota [toponym]’ (cf. e.g. CKD 637 o1 *deviyae Khotaṃnaṃmi ukasta* ‘the queen went away to Khotan [toponym]’). 345 uo2; 351 o4; 362 o4, o5; ***Calmadanaṃmi*** (*Calmatanaṃmi*), toponym: CKD 4 uo2, uo3; 122 o3; 254 o4; 309 o2, o3; 324 + 328 uo2; 329 o3, o4, o5; ***ñanaṃmi* ‘**knowledge’: CKD 83 uo3; 247 o3; 385 o3; ***tarmaṃmi***, a type of location: CKD 125 r1; ***Tsag̱aṃmi*** (*Tsagaṃmi*), toponym: CKD 68 uo3; 90 o1; 255 o1; ***daśaṃmi*** ‘ten [also as an administrative unit]’: CKD 170 oA1; 341 o3; ***divasaṃmi*** ‘day’: CKD 106 uo8; 123 o2; 340 o2; ***deśaṃmi*** ‘land’: CKD 55 uo3; 272 o6; 331 cr2; ***draṃgaṃmi*** (*traṃghami*) ‘office’: CKD 98 o2; 173 oA1; 283 o7; 357 o5 (2×), o6; ***nagaraṃmi*** (*nagaṃraṃmi*) ‘city’: CKD 25 o2; 86 r5; 133 o2; 272 o3, o5, o7, o8; 296 cr1; ***nadhami*** ‘bundle’: CKD 52 uo3; ***Navoteyaṃmi***, toponym: CKD 351 o4; ***nac̄iraṃmi*** ‘hunting’: CKD 156 uo1–2; ***Ninaṃmi***, toponym: CKD 14 cr1; 189 o2 (2×), o3, r1; ***paṃcami*** ‘five’: CKD 329 o5; ***paṃcadaśami*** ‘fifteen’: CKD 368 o4; ***paṃthaṃmi*** (*paṃthami*) ‘road’: CKD 165 o5; 359 r4; ***padami*** ‘foot’: CKD 339 o2; ***padamulaṃmi*** ‘root of the feet’: CKD 77 o2; 89 o3; 104 r1; 107 o2; 127 o2; 130 uo1; 139 o2; 140 co2, uo2; 164 o2; 203 o3; 206 cr3; 207 o3; 216 o3; 247 o1; 248 o2; 249 o2; 259 o1; 272 o2; 288 co2, uo2; 289 o2; 291 o3; 292 o2; 314 o1–2; 320 o3; 329 o2–3; 333 o2; 341 o2; 349 o3; 351 o3; 357 o2; 358 o2; 370 co2; 385 o1; 390 o2; ***parvataṃmi*** ‘mountain’: CKD 231 r2; ***pirovaṃmi*** (*pirovami*) ‘fort’: CKD 122 o4; , o4; ***Pis̱aliyaṃmi*** (*Pis̱alýiyami*), toponym: CKD 122 o2; 291 o5; ***puṣg̱araṃniyaṃmi*** *,* a type of location 70
Burrow’s (1937: §49, pp. 19–20) etymology from OIA *puṣkara*- ‘pond’ is phonologically implausible. : CKD 383 r7; ***potg̱eyaṃmi***, a type of water reservoir: CKD 120 o1; 204 o1; 397 o2, o3, o4; ***pradej̱ami*** (*padej̱ami*; *pradeśaṃmi*; *pradeśami*) ‘region’: CKD 41 r1; 146 oC1; 148 oA1; 163 r1; 168 o2, r1; 179 oA1, oA2, oB1, oB2; 242 rA1, rD1; 271 + 338 uo6; 337 oA1, oB1; 398 o1; ***pravaṃnag̱aṃmi*** ‘document’: CKD 59 r2–3; ***prastami*** ‘elevated ground’: CKD 225 r5; ***Buṃniyaṃmi***, toponym: CKD 157 r1; ***bhumaṃmi*** (*bhumami*) ‘land’: CKD 222 + 336 uo6; 225 o4, o7; 278 r4; 331 uo3; ***maṃnuśami*** ‘man’: ; ***manasaṃmi*** (*maṃnasaṃmi*; *manasiyaṃmi*) ‘mind’: CKD 68 cr3; 217 r2; 358 o9; 367 o4; 399 rA2; ***masaṃmi*** ‘month’: CKD 119 o5; ***Masinaṃmi***, toponym: CKD 278 r6; 374 o2; ***masuaṃmi*** ‘wine’: CKD 291 o5; 333 o6; ***miṣiyaṃmi*** ‘agricultural land’: CKD 90 o2; 212 uo2; 327 o3; ***muliyaṃmi*** ‘price’: CKD 345 uo10; ***yaṃñami*** ‘sacrifice’: CKD 195 + 726 uo4; ***rajaṃmi*** (*rajyami*) ‘state, province’: CKD 40 uo2, uo4; 109 r3; 152 uo4; 182 o4; 198 o2 (2×); 229 r1; 272 o4; 368 o3; ***rayakaṃmi*** ‘royal (office)’: CKD 345 cr4; ***rayadvaraṃmi*** (*rayatvaraṃmi*; *rajadvaraṃmi*; *dvaraṃmi*) ‘royal palace’: CKD 3 cr1; 5 cr2; 6 cr1; 7 cr2; 11 cr1–2; 18 uo3; 27 cr2; 33 uo4; 35 r3; 37 cr2; 45 uo2, uo4, cr2; 46 cr2, cr4; 47 uo3; 49 cr1; 53 cr1; 61 cr1; 62 co3; 63 uo3; 68 cr1; 71 cr4; 83 uo4, uo6; 124 cr1; 144 co3; 155 r1; 159 r6–7; 175 o1; 180 oB4, oB5; 200 o3; 201 o4; 216 r6; 217 r1; 223 cr3; 234 o2; 235 cr2; 240 r2; 256 cr2; 272 o7, o8, o11; 273 r1; 286 o3; 295 o3; 296 cr1; 297 uo4, cr1, cr3; 298 r3; 311 r1; 312 cr1, cr2; 344 r1; 345 uo6, cr3; 347 r3; 352 r1; 356 o3; 357 o5; 358 o4; 359 r1; 364 r1, r3; 386 o3; 387 o5, o7; 393 o4; 399 o5; ***Remenaṃmi***, toponym: CKD 214 o4; 251 o3; 376 o1; ***laṭhanami***, a type of land: CKD 392 o4; ***livistaraṃmi*** ‘detailed writing’: CKD 165 o9; 375 o3; ***lekhaṃmi*** ‘letter’: CKD 144 co4; 308 o4; ***Lominanaṃmi***, toponym: CKD 122 o1; ***varṣaṃmi*** (*varṣami*) ‘year’: CKD 15 uo3; 162 uo4, uo5; 226 o3; 272 o5; 305 o6; 310 uo2; 387 o9; ***varṣavasaṃmi*** ‘rainy season’: CKD 211 r6; ***varṣaśadami*** ‘a hundred years’: CKD 348 uo6; ***vivataṃmi*** ‘dispute’: CKD 364 r4; ***viṣeyaṃmi*** ‘territory’: CKD 292 o4; ***Vr̥ganic̄itaṃmi***, toponym: CKD 116 o1; ***śakasyami*** *,* a type of location: CKD 12 uo2; ***śataṃmi*** (*śadami*) ‘hundred [an administrative unit]’: CKD 41 o1 (2×), o2 (3×), o3, r1; 46 cr2, cr3; 73 oC1; 74 oA1, oB1, rA1, rB4; 76 oA2, oA3, oA4, oA5, oA6, oA7, oA8; 92 o1 (2×); 168 o2; 169 rA1, rB1, rC1, oA2, oB1, oC1, oD1, oE1; 170 r1; 173 oA1, oA2, oA3, oA4, oA5, oA6, oA7; 174 oA1; 185 oA1, oB1, oC1, oD1; 221 o1; 268 r1; 299 o1; 313 oA1; 342 oA1, oA5, oA6, oC1, oC2; ***śarataṃmi*** (*śaradaṃmi*) ‘autumn’: CKD 5 uo2, cr1 (2×); 109 r2; 198 o3; 236 uo2; 283 o4; 358 o3; ***ṣilýog̱aṃmi*** ‘written document’: CKD 140 cr1; 359 r2; ***Sacaṃmi*** (*Sācaṃmi*; *Sachaṃmi*), toponym: CKD 14 uo3; 123 o2, o3; 159 r2; 160 o5; 368 o4, o5; ***sasteyaṃmi*** (*s̱asteyaṃmi*) ‘day’: CKD 153 r2; 329 o5; ***simaṃmi*** ‘border’: CKD 163 r2; 367 o2; ***S̱armanaṃmi***, toponym: CKD 157 r1; ***hastaṃmi*** (*hastami*) ‘hand’: CKD 4 uo3; 21 uo3; 23 o3; 28 o3; 42 cr1; 59 r2; 70 cr1; 83 cr4; 100 o1, o4 (2×); 106 cr5; 128 o6; 136 uo2; 140 uo6, cr4; 143 uo2; 159 r2; 164 o8; 165 o3, o12; 177 r5; 211 r8; 214 o2; 217 r1; 247 o4 (2×); 257 r1; 263 r2; 272 o3; 288 cr3; 292 o2–3; 309 o3; 310 uo3 (3×), uo4, urB2, urB3; 311 r1; 320 r3; 329 o3–4; 333 o6; 341 o3; 357 o3; 358 o2–3; 361 o1, o2; 368 o4; 376 o7; 379 o3, o4; 385 o6; 387 o3, o11; ***hemaṃtaṃmi*** ‘winter’: CKD 211 r4; 309 o2 **∼ 85 types; 545 tokens.**

#### b. Nominal attestation of the loc.pl in -*eṣu* 71 I leave aside the badly preserved /// *ciyeṣu* in CKD 484.

***uṭiyeṣu*** ‘she-camels’: CKD 134 o2; ***karyeṣu*** ‘works’: CKD 107 o6; 821 o2; ***goṭheṣu*** ‘farms’: CKD 547 o3; ***Calmatāneṣu*** ‘the people of Calmadana’: CKD 119 o4; ***nagareṣu*** ‘cities’: CKD 364 r4; ***nagaradraṃgeṣu*** ‘offices of the city’ CKD 272 o6; ***parvateṣu* (*parvadeṣu*)** ‘mountains’: CKD 133 o4; 637 o3, o10; ***paśuveṣu*** ‘sheep’: CKD 568 uo4–5; ***posathakarmanimaṃtreṣu*** ‘invitations to the *posatha* ceremony’: CKD 489 r7–8; ***pr̥ṭheṣu*** ‘backs’: CKD 400 o2; ***bahirneṣu*** ‘outside’: CKD 568 uo3; ***muṣg̱eṣu***, unidentified body part (cf. Schoubben 2024: 303 fn. 687): CKD 540 uo2; ***yaṃñeṣu*** ‘sacrifices’: ; ***varṣeṣu*** ‘years’: CKD 256 uo3, uo5, cr1; 819 uo5; ***viṣeyeṣu*** ‘territories’: CKD 621 o4; 842 o6; ***Sāceṣu*** ‘the people of Saca’: CKD 637 o10; ***hastalekheṣu*** ‘hand-documents’: CKD 788 cr4; 814 3. **∼ 17 types; 25 tokens.**
